# Socio-environmental predictors of diabetes incidence disparities in Tanzania mainland: a comparison of regression models for count data

**DOI:** 10.1186/s12874-024-02166-w

**Published:** 2024-03-26

**Authors:** Sauda Hatibu Mbwambo, Maurice C. Mbago, Gadde Srinivasa Rao

**Affiliations:** 1 Department of Statistics, Dar es Salaam, University of Dar es Salaam, P.O. Box 35047, Dar es Salaam, Tanzania; 2 Department of Mathematics and Statistics, The University of Dodoma, P.O. Box 338, Dodoma, Tanzania

**Keywords:** Diabetes, Socio-environmental factors, Count data models, Generalized Poisson, Tanzania

## Abstract

**Background:**

Diabetes is one of the top four non-communicable diseases that cause death and illness to many people around the world. This study aims to use an efficient count data model to estimate socio-environmental factors associated with diabetes incidences in Tanzania mainland, addressing lack of evidence on the efficient count data model for estimating factors associated with disease incidences disparities.

**Methods:**

This study analyzed diabetes counts in 184 Tanzania mainland councils collected in 2020. The study applied generalized Poisson, negative binomial, and Poisson count data models and evaluated their adequacy using information criteria and Pearson chi-square values.

**Results:**

The data were over-dispersed, as evidenced by the mean and variance values and the positively skewed histograms. The results revealed uneven distribution of diabetes incidence across geographical locations, with northern and urban councils having more cases. Factors like population, GDP, and hospital numbers were associated with diabetes counts. The GP model performed better than NB and Poisson models.

**Conclusion:**

The occurrence of diabetes can be attributed to geographical locations. To address this public health issue, environmental interventions can be implemented. Additionally, the generalized Poisson model is an effective tool for analyzing health information system count data across different population subgroups.

## Background

To date, non-communicable, diseases including diabetes, are still a global health challenge affecting people of all ages; however, elderly people are at higher risk [1, 2]. In 2016, statistics showed that Non-Communicable Diseases (NCDs) were responsible for 80% of all deaths worldwide. The NCD death risk is notably higher in Sub-Saharan Africa, Central Asia, and Eastern Europe [3]. In Tanzania, as in Sub-Saharan Africa, there is evidence of a high prevalence of NCD cases, including diabetes [4, 5].

The emergence of NCDs in humans is influenced by a complex combination of various factors, which include environmental conditions, cultural beliefs, self-management, socio-demographic factors, genetics, and biology [1]. These diseases are sometimes referred to as behavioural diseases because, apart from other factors, self-management, which is linked by a person’s behavioural practice in running his/her daily life can increase one’s likelihood of developing NCDs [1, 6]. Cultural norms and values can also influence human behaviour, resulting in regional and national variations in the prevalence of NCDs [7]. This study aims to investigate the impact of socio-environmental factors, which are the council’s zone and residence, along with other factors on the total diabetes incidences in the council. The link between environment and human behaviours is well explained in some behavioural theories, including the reciprocal deterministic concept of social cognitive theory [8].

Currently, many scenarios in public health and official statistics include count data. Count data include specific disease cases reported in a particular geographical unit, the total number of fatalities occurring within a given timeframe, etc. The Poisson model is a well-known method for modeling count data and has been applied in many situations [9–14]. However, it assumes that the subject occurs randomly and at a constant rate, resulting in equality mean and variance, which is often unrealistic in real-life situations. When data exhibit over-dispersion, the negative binomial (NB) model is often used as an alternative to the Poisson model [11, 15, 16]. Occasionally, under dispersion also occurs among count data, especially for rare events. To tackle this issue, researchers have developed new models that can model count data that exhibits over, under, or equal dispersion. These models were obtained as a result of generalization or mixing with the Poisson model. Examples of these models are the Generalized Poisson (GP) [17], the Weighted Poisson, the Conway-Maxwell-Poisson (CMP), the Hyper-Poisson (HP) [18], Extended Bi-parametric Waring (EBW) [19], and the Complex Tri-parametric Pearson (CTP) [20].

Many of the distributions mentioned above have complex functional forms, which can lead to significant computational challenges and make them difficult to use. For this reason, the GP model was selected for this study. This model has a well-defined functional form and allows easy parametric estimation [21–24]. The GP model is the best option to be used in health and behavioural studies, for many reasons, including the non-uniformity of the population being studied, where individuals tend to cluster or aggregate within a particular combination with similar characteristics; dependence among observations due to environmental factors, where there are high incidences of diabetes cases in the same geographic area due to similarity in socio-cultural factors; which causes unequal dispersion which happens in the data [15, 21].

Numerous studies on NCDs, including diabetes, have been conducted [4, 5, 25–29]. However, none have utilized the GP model or quantified socio-environmental factors (such as zone and council residence) in NCD occurrences in mainland Tanzania. This study aims to establish a model that can be adopted in modeling NCDs count incidences associated with socio-environmental and other risk factors. Hence, it emphasizes environmental-based approaches to eradicating NCDs in Tanzania, and the model can also be adopted in similar scenarios. Many research articles elaborate on the application of GP regression in modeling over-dispersed data [24, 30–35]. However, the articles do not describe or quantify how overestimation of the standard error occurs when using standard Poisson in modeling over-dispersed data as the current does.

## Methods

### Design and settings

This study utilized cross-sectional reseach design. Secondary data collected by the District Health Information System (DHIS2) and the National Bureau of Statistics (NBS) in 2020 were used for analysis. The response variables represent the number of patients diagnosed with diabetes mellitus admitted to all health facilities within a council, except for regional referral and zonal hospitals. Information collected in 2020 and across 184 councils in Tanzania mainland is used in this study.

### Models description

Generalized linear models (GLMs) extend linear models (LMs) when the response variable is not normally distributed, allowing for the representation of non-normal response variables. In GLMs, the distribution of the response variable can be counted, categorical, discrete, ordinal, and many others as long as it belongs to the exponential family of distributions. This family has several well-known distributions including the Poisson distribution and its generalization, the binomial distribution, Gamma distribution, and many others. GLMs can be described using the following equation:

$$g\left(\mu \right)={X}_{i}^{T}\beta$$

And is mainly characterized by its three components: (1) A random component, which describes the outcome variable ${Y}_{i}$ of the ${i}^{th}$ observation by its probability density function. (2) A linear component ${X}_{i}^{T}\beta$, where ${X}_{i}^{T}$ is the vector of predictors and $\beta$ is a column vector of model coefficients. (3) Differentiable link function $g\left(\mu \right)$, which relates the mean of the response variable and the linear function of the predictor variables [9, 36].

The Poisson process is often used to explain the variations in count data compared to a predicted average [9]. However, this model has certain assumptions, including that the data must be equally distributed and that the mean must always equal the variance. Poisson regression is a well-known model for modeling the means of $n$ non-negative count response variables ${y}_{1},{y}_{2},\dots ,{y}_{2}$. Let ${Y}_{i} i=\text{0,1},\dots .$, be the response variable which represents the number of diabetic patients admitted to specific council in 2020 and ${X}_{i}^{{\prime }}={X}_{1i},\dots ,{X}_{ki}$ represents a k-dimension vector of linear predictors associated with the response variable $Y$. A Poisson regression of the response variable given predictors is written as:

1 $$P\left({Y}_{i}={y}_{i}/{X}_{i}={x}_{i}\right)=\frac{{e}^{-{\mu }_{i}}{{\mu }_{i}}^{{y}_{i}}}{{y}_{i}!}; {y}_{i}=\text{0,1},\dots$$

For Poisson distribution, we have $E\left({Y}_{i}={y}_{i}/{X}_{i}={x}_{i}\right)={\mu }_{i}=Var\left({Y}_{i}={y}_{i}/{X}_{i}={x}_{i}\right)$

The logarithm of the likelihood of the equation above can be written as:

$$\sum\limits _{i=1}^{n}\left[{y}_{i}ln{\mu }_{i}-{\mu }_{i}-ln{y}_{i}!\right]$$

By substituting ${\mu }_{i}={e}^{{{x}_{i}}^{{\prime }}\beta }$, we obtain the logarithm of the likelihood function in terms of $\beta^{\prime }s$ which can be written as:

$$\sum\limits_{i=1}^{n}\left[{y}_{i}{{x}_{i}}^{{\prime }}\beta -\text{exp}\left({{x}_{i}}^{{\prime }}\beta \right) -ln{y}_{i}!\right]$$

Maximum likelihood estimates of β’s can be obtained by differentiating the logarithm of the likelihood equation with respect to β’s and setting the results equal to zero.

Thus, the Poisson regression model of the mean parameter ${\mu }_{i}$ is written as

$$ln\mu_i={x_i}^{\prime}\underline\beta$$

$$ln{\mu }_{i}={\beta }_{0}+{\beta }_{1}{x}_{i1}+\dots +{\beta }_{k}{x}_{ik}={\beta }_{0}+\sum\limits_{j=1}^{k}{\beta }_{j}{x}_{ij}$$

$${\mu }_{i}=exp\left({\beta }_{0}+\sum\limits_{j=1}^{k}{\beta }_{j}{x}_{ij}\right)$$

As data are collected from councils across various geographical locations, including areas with differing behavioural patterns, there is a high probability of unequal dispersion in the data. This suggests that the data may have under- or over-dispersion. If Poisson regression is used to model these data, it could lead to incorrect conclusions because the standard error may be overestimated [15, 37]. The negative binomial model, also known as the Poisson Gamma mixture, is considered a better alternative to Poisson regression when dealing with over-dispersed count data. The model’s mean and variance have a quadratic relationship, resulting in its being named NB2 [11].

The NB model was formulated as an extension of the Poisson model by considering the idea that the modeled outcomes cannot happen at a constant rate, leading to heterogeneity in the outcomes. The extended model can be formulated as follows:

A negative binomial distribution is generated using a series of Bernoulli trials with a constant success probability p. Let Y be the number of attempts that failed before the ${k}^{th}$ success $(k>0)$, then, Y follows a negative Binomial distribution with probability mass function (pmf) written as follows:

2 $${p}_{Y}\left(y\right)=\left(\begin{array}{c}k+y-1\\ y\end{array}\right){p}^{k}{\left(1-p\right)}^{y};y=\text{0,1},2,\dots$$

The mean and variance of Y are $\frac{pk}{\left(1-p\right)}$ and $\frac{pk}{{\left(1-p\right)}^{2}}$ respectively. In the negative binomial regression model the interest in modeling the mean of the outcome variable Y with its realization ${y}_{1},{y}_{2},\dots ,{y}_{2}$, and ${X}_{i}^{{\prime }}={X}_{1i},\dots ,{X}_{ki}$ denotes the matrix of predictors. Parametrization of Eq. (2) above in terms of $\mu$ and dispersion parameter $\alpha$ yield NB regression model as described below:

Let $p=\frac{\alpha }{\alpha +\mu }$, where $\alpha =k$, furthermore it is known that $$\left(\begin{array}{c}\alpha +{y}_{i}-1\\ {y}_{i}\end{array}\right)=\frac{\left(\alpha +{y}_{i}-1\right)!}{{y}_{i}!\left(\alpha -1\right)!}=\frac{{\Gamma }\left(\alpha +{y}_{i}\right)}{{y}_{i}!{\Gamma }\left(\alpha \right)}$$, then the pmf of Y in Eq. (2) can be written as:

3 $$f\left({Y}_{i}={y}_{i}/{X}_{i}={x}_{i}\right)=\frac{{\Gamma }\left(\alpha +{y}_{i}\right)}{{y}_{i}!{\Gamma }\left(\alpha \right)}{\left(\frac{{\mu }_{i}}{\alpha +{\mu }_{i}}\right)}^{{y}_{i}}{\left(\frac{\alpha }{\alpha +{\mu }_{i}}\right)}^{\alpha };y=\text{0,1},2,\dots$$

where ${\Gamma }$ represents the gamma function and $\alpha$ is a dispersion index that has been modified to take positive values only. The NB can also be obtained by using the Poisson mixture gamma formula. Then, $Y\sim NB\left(\mu,\alpha\right)$ and the mean and variance of Y are ${\mu }_{i}$ and ${\mu }_{i}+\frac{{{\mu }_{i}}^{2}}{\alpha }$ respectively. When $\alpha \to \infty$, the mean and variance of Y tend to be equal, which implies that the Poisson model is a special case of the negative binomial model [9, 11, 15, 36, 38].

The likelihood of Eq. (3) is proportional to:

$$\prod\limits_{i=1}^{n}f\left({Y}_{i}={y}_{i}/{X}_{i}={x}_{i}\right)=\prod\limits_{i=1}^{n}\frac{{\Gamma }\left(\alpha +{y}_{i}\right)}{{y}_{i}!{\Gamma }\left(\alpha \right)}{\left(\frac{{\mu }_{i}}{\alpha +{\mu }_{i}}\right)}^{{y}_{i}}{\left(\frac{\alpha }{\alpha +{\mu }_{i}}\right)}^{\alpha }$$

It is known that:

$$\frac{{\Gamma }\left(\alpha +{y}_{i}\right)}{{\Gamma }\left(\alpha \right)}=\alpha \left(\alpha +1\right)\dots \left({y}_{i}-1+\alpha \right)$$

It follows that:

$$ln\left(\frac{{\Gamma }\left(\alpha +{y}_{i}\right)}{{\Gamma }\left(\alpha \right)}\right)=ln\left(\alpha \left(\alpha +1\right)\dots \left({y}_{i}-1+\alpha \right)\right)=\sum\limits_{k=0}^{{y}_{i}-1}ln\left(\alpha +k\right)$$

and the log-likelihood is given by:

$$=\sum\limits_{i=1}^{n}\left(\sum\limits_{k=0}^{{y}_{i}-1}ln\left(\alpha +k\right)-ln{y}_{i}!+{y}_{i}ln{\mu }_{i}-{y}_{i}ln\left(\alpha +{\mu }_{i}\right)+\alpha ln\alpha -\alpha ln\left(\alpha +{\mu }_{i}\right)\right)$$

It is known that

$$\mu_i=\left({exp_i}^{\prime}\underline\beta\right)$$

$$l(\beta ,\alpha )=\sum\limits_{i=1}^{n}\left(\sum\limits_{k=0}^{{y}_{i}-1}ln\left(\alpha +k\right)-ln{y}_{i}!+{y}_{i}ln{\mu }_{i}+\alpha ln\alpha -{(y}_{i}+\alpha )ln\left(\alpha +{\mu }_{i}\right)\right)$$

Estimates of the regression coefficients $\beta^{\prime }s$ and dispersion index $\alpha$ are obtained by substituting into the above equation and differentiating it with respect to $\beta^{\prime }s$ and $\alpha$ and setting the result equal to zero.

Then, the negative binomial regression model can be written as:

$${ln\mu }_{i}={\beta }_{0}+\sum\limits_{j=1}^{k}{\beta }_{j}{x}_{ij}$$

NB model cannot be used to model equal and under-dispersed data. The finding reveals that the NB model faces convergence issues if inappropriately used to model count data, which does not exhibit over-dispersion [38].

Many articles use the latest count model generalizations; however, the GP model remains beneficial and user-friendly [39]. This model can model stochastic processes with count data that have equal, under, or over-dispersion. Moreover, estimating the parameters of this model is simple compared to other generalized models. Due to the reasons mentioned above, this study employs the model introduced by Consul and Jain [17, 40]. Let ${Y}_{i}$ represent diabetes incidences for inpatient recorded in a certain council for 2020. Then, ${Y}_{i}$ represents the response variable having response values $y_1,y_2,\dots\;\dots,y_n$ associated with several explanatory variables. Then, ${Y}_{i}$ follows a GP distribution, and its probability mass function can be written as:

3 $$\left(y;\alpha,\delta\right)=\left\{\begin{array}{lc}\frac{{\alpha\left(\alpha+\delta y\right)}^{y-1}}{y!}exp\left(-\alpha-\delta y\right);&y=0,1,2\dots\;\dots\;\dots\\0;&For\;y>m\;when\;\delta\;<0\end{array}\right.$$

The mean and variance of the GP distribution are $\mu =\frac{\alpha }{\left(1-\delta \right)}=\vartheta \alpha$ and $Var\left(y\right)={\frac{\alpha }{{\left(1-\delta \right)}^{3}}=\vartheta }^{2}\mu$ respectively where $\delta$ is called the dispersion parameter expressed by the dispersion factor $\vartheta =\frac{1}{\left(1-\delta \right)}$.

If $\delta =0$, GP distribution reduces to the standard Poisson distribution when $\delta <0$, it represents under-dispersion, and if $\delta >0$, it represents over-dispersion.

Suppose explanatory variables are represented by $\left(K-1\right)$ dimensional vector ${X}_{i}^{{\prime }}={X}_{1i},\dots ,{X}_{ki}$. The conditional distribution of ${Y}_{i}$ for a given value of ${x}_{i}$ follows a GP distribution with the mean value given by:

$$E\left({Y}_{i}/{x}_{i}\right)=\mu \left({x}_{i}\right)=\frac{{\alpha }_{i}}{\left(1-{\delta }_{i}\right)}={C}_{i}f\left({x}_{i},\beta \right)$$

where $f\left({x}_{i},\beta \right)>0$ represents a differentiable function, ${C}_{i}$ represents a measure function and $\underline\beta$ is the K-dimensional vector of regression parameters.

From the mean of GPD, $\mu =\frac{\alpha }{\left(1-\delta \right)}$ and $\vartheta =\frac{1}{\left(1-\delta \right)}$ the dispersion factor, then the generalized Poisson regression can be deduced as

4 $$P\left(Y=y/x\right)=\left\{\begin{array}{lc}\mu\left[\mu+\left(\vartheta-1\right)y\right]^{y-1}\frac{\vartheta^{-y}}{y!}exp\left[-\frac1\vartheta(\mu+\left(\vartheta-1\right)y)\right];&y=0,1,2\dots\;\dots\\0;&For\;y>m\;when\;\vartheta<1,\end{array}\right.$$

where, $\mu =\mu \left(x\right)>0$, $\vartheta \ge \text{max}(^{1}\!\left/ \!_{2}\right.,1-^{\mu }\!\left/ \!_{4}\right.)$, $\sqrt{\frac{var(y/x)}{\mu (y/x)}}$ stands for the square root of the dispersion index, and *m* is the largest positive integer for which $\mu +m\left(\vartheta -1\right)>0$ when $\vartheta$ is non-negative.

When $\vartheta =1$, GP distribution is condensed to standard Poisson regression (proper in modeling equal dispersed data); when $\vartheta >1$ GPR is appropriate in modeling over-dispersed data, and when $\vartheta <1$, GPR is used to fit under-dispersed data [34, 40].

Similar to the standard Poisson regression model, GPR uses a log link to connect the mean of the response variable and explanatory variables, as shown below:

5 $$\mu=\mu\left(x\right)=exp\left(\underline x_i^T\underline\beta\right)\;or\;\log\;\mu\left(x\right)=\left(\underline x_i^T\underline\beta\right)$$

where, $\mu =\mu \left(x\right)=\frac{\alpha }{\left(1-\delta \right)}$ is the mean, $\underline x_i^T$ represents the $(k-1)$ dimensional vector of explanatory variables and $\underline\beta$ is the k-dimensional vector of regression parameters.

In this study, diabetes counts in the council in 2020 have been used as a response variable regressed to the following elaborated explanatory variables:

**Table:** 

S/no	Variable name	Variable description
1	${X}_{1}$	Percentage of people living with HIV (PPLHIV) in the year 2020 in a specific council
2	${X}_{2}$	Council’s 2020 population projection
3	${X}_{3}$	Council’s estimated gross domestic product (GDP) at market price
4	${X}_{4}$	Council’s residence (Rural or urban)
5	${X}_{5}$	Number of health facilities in the council
6	${X}_{6}$	Percentage of males in total diabetes count
7	${X}_{7}$	Zone (Lake, Southern, Southern Highlands, Central, Eastern, Northern, and Western zones)

The model can be written as:

6 $$\mu=exp\left(\underline x_i^T\underline\beta\right)$$

$$\underline x_i^T=\left[1\;x_{1i}\;x_{2i}\;x_{3i}\;x_{4i}\;x_{5i}\;x_{6i\;}x_{7i}\right]$$

$$\beta^T=\left[\beta_0\;\beta_1\;\beta_{2\;}\beta_3\;\beta_4\;\beta_5\;\beta_6\;\beta_7\right]$$

Then,

$$exp\left(\underline x{_i^T\underline\beta}\right)=exp\left(\beta_0+\beta_1x_{1i}+\beta_2x_{2i}+\beta_3x_{3i}+\beta_4x_{4i}+\beta_5x_{5i}+\beta_6x_{6i}+\beta_7x_{7i}\right)$$

Estimation of model coefficients $\underline\beta$ was performed through the maximum likelihood method. Additionally, the goodness of fit of the GP model over the NB and Poisson models is also evaluated using AIC, AICc, and BIC.

## Results

**Fig. 1:**
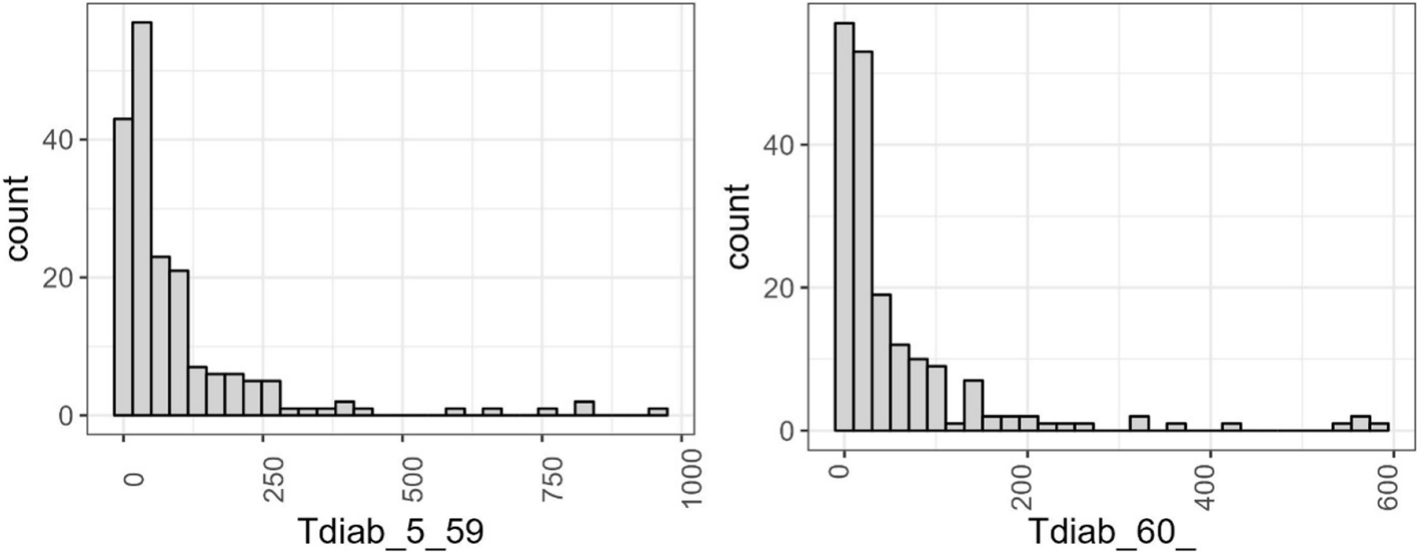
Histogram showing diabetes counts per age group

The histogram in Fig. 1 describes the dispersion property of diabetes incidence across councils. The plot indicates a significant positive skew, with more small numbers, including zero, and few large counts, suggesting overdispersion among diabetic patients incidence between two age groups, namely, age 5 to 59 and 60 or older. This is common among disease incidence datasets since sometimes disease severity is triggered by behavioural patterns among subpopulations being sampled, which vary from one society to another, leading to unequal dispersion. Since the data used reveals unequal dispersion, the GP model may give a precise estimate with meaningful inference [19, 20, 24, 39, 40].

**Fig. 2:**
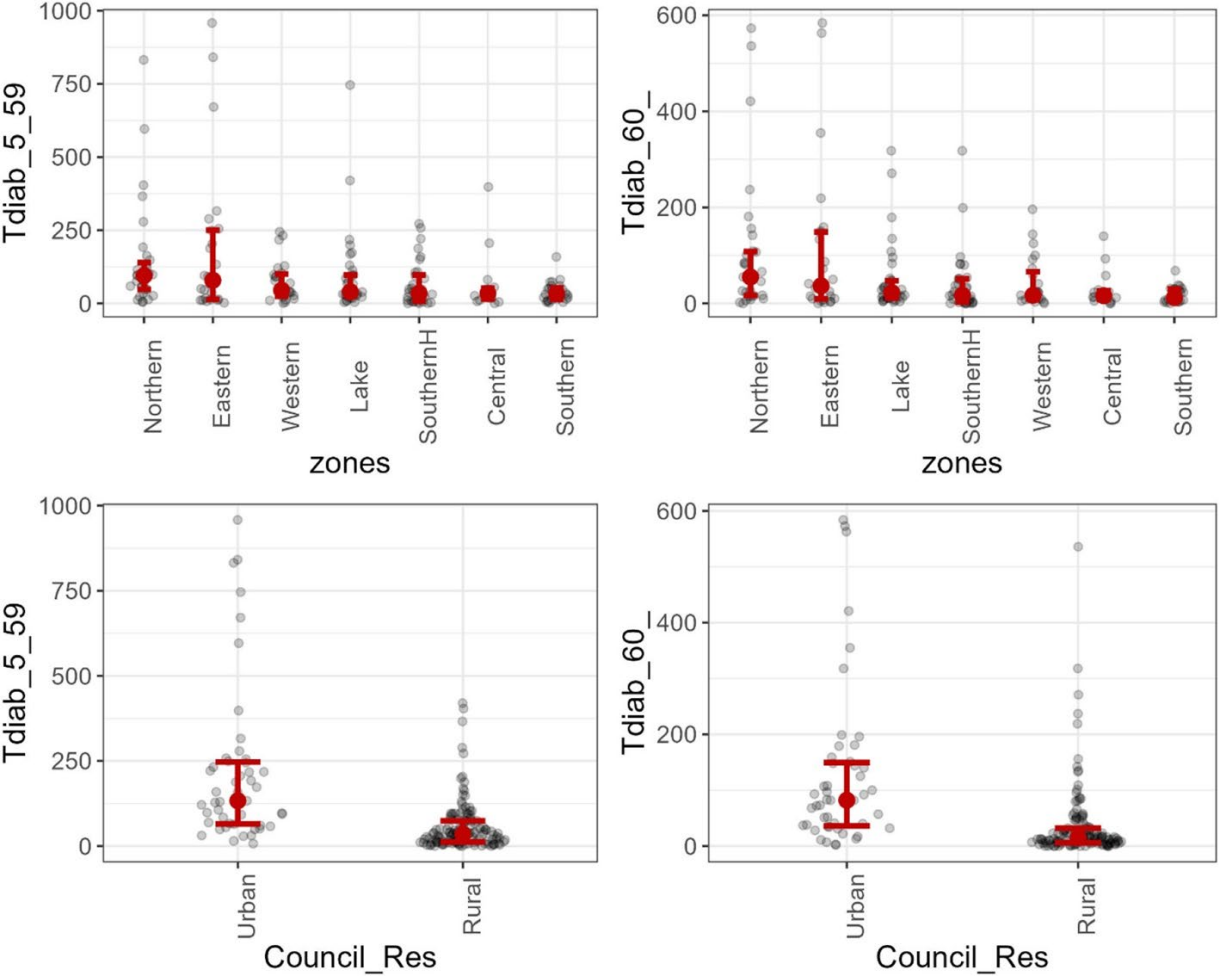
Beeswarm plots of the distribution of diabetes counts within councils by environmental location

The beeswarm plots in Fig. 2 display diabetes records per geographical location. The plots indicate a concentration of diabetes in areas with similar traits. Categories are arranged in ascending order based on the number of diabetes cases reported. The councils in the northern zone have more diabetes cases than the other zones, while the councils in the southern zone have fewer counts than other zones. Furthermore, councils inside high-count zones record fewer zeros and low counts than councils within low-count zones. Additionally, there is a significant difference in diabetes records between rural and urban councils, with rural councils record many zero and small incidents while urban councils record a substantially large number of diabetes cases.

**Table 1:** Distribution of diabetes counts for patients aged 5–59 years within councils and associated environmental predictors

Covariates	Level	Diabetes counts for patients aged 5–59 years			*P* value
		<50	51–200	>200	
Council’s residence	Rural	93 (50.54)	38 (20.65)	8 (4.34)	< 0.001
	Urban	7 (3.8)	22 (11.96)	16 (8.70)	
Zones	Central	11 (5.98)	2 (1.09)	2 (1.09)	0.01106
	Eastern	11 (5.98)	5 (2.72)	8 (4.35)	
	Lake	21 (11.41)	12 (6.52)	3 (1.63)	
	Northern	9 (4.89)	17 (9.24)	5 (2.72)	
	Southern	16 (8.70)	7 (3.8)	0 (0.0)	
	Southern Highlands	20 (10.87)	9 (4.89)	3 (1.63)	
	Western	12 (6.5)	8 (4.3)	3 (1.6)	

**Table 2:** Distribution of diabetes counts for patients aged 60 years and above within councils and associated environmental predictors

Covariates	Level	Diabetes counts for patients aged 60 years and above			*P* value
		<50	51–200	>200	
**Council’s residence**	Rural	112 (60.87)	21 (11.41)	6 (3.26)	< 0.001
	Urban	16 (8.70)	24 (13.04)	5 (2.72)	
**Zones**	Central	12 (6.5)	3 (1.6)	0 (0.0)	0.00474
	Eastern	14 (7.6)	6 (3.3)	4 (2.2)	
	Lake	28 (15.2)	6 (3.3)	2 (1.1)	
	Northern	13 (7.1)	14 (7.6)	4 (2.2)	
	Southern	22 (12.0)	1 (0.5)	0 (0.0)	
	Southern High	23 (12.5)	8 (4.3)	1 (0.5)	
	Western	16 (8.7)	7 (3.8)	0 (0.0)	

Tables 1 and 2 summarize the association between diabetes count categories and categorical predictors in Tanzania mainland for patients with 5–59 and 60 years and above age groups respectively. The dataset consists of diabetes records from 184 councils, with the minimum and maximum recorded numbers being 0 and 958, respectively, across two age groups. The chi-square test for dependence was used to measure the presence of a statistically significant association between diabetes count and two categorical predictors associated with environmental factors: council residence (rural or urban) and council zone (northern, eastern, lake, southern, southern highland, western, and central zones) among datasets from two distinct age groups. For both scenarios, the *p*-value is less than 0.001, indicating the presence of an association among categories.

Moreover, Tables 1 and 2 demonstrate larger counts of diabetes incidences recorded among councils located in urban areas than councils in rural areas in diabetes patients aged 5–59 years. The presence of large numbers of diabetes records among people aged 5–59 years indicates the high chance of premature mortality and morbidity due to diabetes contrary to sustainable development goal number 3.4. For 60 years and older age groups, 60.8% of councils located in rural areas recorded fewer than 50 diabetes patients whereas 8.7% of councils in urban areas recorded fewer than 50 patients. Moreover, in the southern zone, none of the councils recorded more than 200 diabetes cases among both age groups.

**Table 3:** Model fit results from generalized poisson, negative binomial, and standard poisson models

Model	Coefficients:	Estimate	Std. Error	*z* value	Pr(>|z|)
Generalized Poisson Regression	(Intercept):1	3.5400	0.3080	11.492	< 2e-16
	(Intercept):2	1.4443	0.0403	35.815	< 2e-16
	PPLHIV	0.0616	0.0241	2.556	0.0106
	Pop	0.2264	0.0285	6.175	1.98e-15
	GDP	-0.1507	0.0306	-4.922	8.57e-07
	No. of H.facilities	0.0132	0.0034	3.852	0.000117
	% of men	-0.0017	0.0050	-0.343	0.7314
	Council residence (ref.=Rural)				
	Urban	1.1720	0.1067	10.979	< 2e-16
	Council zone (ref.=Northern zone)				
	Central	-0.8480	0.2145	-3.953	7.72e-05
	Eastern	-0.6483	0.1764	-3.675	0.000237
	Lake	-0.7265	0.1495	-4.860	1.17e-06
	Southern	-0.8064	0.1949	-4.138	3.51e-05
	Southern Highland	-0.7467	0.1837	-4.066	4.78e-05
	Western	-0.3392	0.1836	-1.848	0.064655
Negative Binomial distribution	(Intercept):1	3.3498	0.3656	9.163	< 2e-16
	(Intercept):2	0.4740	0.1028	4.613	3.97e-06
	PPLHIV	0.0803	0.0318	2.520	0.01174
	Pop	0.2589	0.0419	6.175	6.61e-10
	GDP	-0.1913	0.0424	-4.508	6.54e-06
	No. of H.facilities	0.0187	0.0046	4.105	4.04e-05
	% of men	-0.0038	0.0059	-0.648	0.516866
	Council residence (ref.=Rural)				
	Urban	1.2570	0.1466	8.572	< 2e-16
	Council zone (ref.=Northern zone)				
	Central	-1.0453	0.2715	-3.850	0.000118
	Eastern	-0.9020	0.2424	-3.722	0.000198
	Lake	-0.9519	0.2053	-4.637	3.54e-06
	Southern	-1.0209	0.2339	-4.364	1.27e-05
	Southern Highland	-0.9335	0.2375	-3.931	8.47e-05
	Western	-0.6395	0.2448	-2.612	0.008989
Poisson regression	(Intercept):1	3.6228	0.05460	66.343	< 2e-16
	PPLHIV	0.0704	0.00403	17.484	< 2e-16
	Pop	0.2122	0.00448	47.350	< 2e-16
	GDP	-0.1800	0.00490	-36.701	< 2e-16
	No. of H.facilities	0.0188	0.00058	32.682	< 2e-16
	% of men	-0.0086	0.00093	-9.239	< 2e-16
	Council residence (ref.=Rural)				
	Urban	1.1996	0.0174	68.764	< 2e-16
	Council zone (ref.=Northern zone)				
	Central	-0.9618	0.03717	-25.876	< 2e-16
	Eastern	-0.7676	0.0279	-27.464	< 2e-16
	Lake	-0.7881	0.0234	-33.706	< 2e-16
	Southern	-1.0425	0.0390	-26.728	< 2e-16
	Southern Highland	-0.8120	0.0312	-26.003	< 2e-16
	Western	-0.4626	0.0320	-14.439	< 2e-16

Table 3 describes the log of expected diabetes counts as a function of selected predictor variables using the GP model (located at the top of the table), the negative binomial model (located in the middle of Table 3), and the standard Poisson (at the bottom of Table 3).

Based on the GP model’s results, the number of diabetes cases in each council is influenced by its population. Thus, more populated councils are anticipated to have more cases of diabetes than the less populated ones. The logs of expected diabetes count in a council would be expected to increase by $0.2264\;(p-value<0.0001)$ when the council’s population increases by one unit. The number of health facilities is significantly associated with the number of diabetes cases in the councils. This may be because the availability of health facilities accelerates disease tracking and recording. Increase in the expected number of health facilities in the councils leads to increase the log of expected diabetes counts by $0.0132\;(p-value<0.0001)$. Moreover, the results suggest positive association between percentage of peoples living with HIV and diabetes incidences in the council. Conversely, GDP per capita shows a significantly negative association with the log of expected diabetes counts in the councils. This implies that diabetes cases happen more in councils with less GDP per capita. On the other hand, there is no significant association between the percentage of male diabetes patients and the number of diabetes cases in the councils.

Predictors representing environmental factors are significantly associated with diabetes counts in the councils. It can be demonstrated that, when other model covariates are held constant, the difference in logs of diabetes counts is predicted to be $1.172\;(p-value< 2\text{e}-16 )$ larger for councils located in urban areas than those in rural areas. Compared to the northern zone, councils located in the central, eastern, lake, southern, and southern highlands zones have a decreased log diabetes counts of $-0.8480\;(p-value=7.72\text{e}-05)$, $-0.6483\;(p-value=0.00024$, $-0.7265\;(p-value=1.17\text{e}-06)$, $-0.8064\;(p-value=3.51\text{e}-05)$, and $-0.7467\;(p-value=4.78\text{e}-05)$ respectively. The reason is because councils in the northern zone contribute more diabetes cases than the other zones. Additionally, there is no significant difference in log diabetes cases in the western zone compared to councils in the northern zone.

There are slight differences between the estimates and standard errors (SEs) obtained by the GP and NB models, resulting in different inferences for the western zone category. The GP model shows that the category’s contribution to the logs of diabetes did not differ from that in the northern zone. In contrast, the NB model shows a significantly decreased log diabetes count by $0.6395\;(p-value=0.00899)$, compared to the northern zone when other factors in the model are kept constant. Additionally, the results in Table 3 indicate that SEs in the Poisson model were underestimated because the values were visually smaller than those obtained in the GP and NB models. This occurs because the Poisson model cannot handle the over-dispersion present in the analyzed datasets. Underestimating SEs leads to incorrect inferences being drawn about some predictors and factors.

Although the GP model finds that one predictor variable (percentage of males hospitalized by diabetes) and one category (western zone) are not important, the NB model only finds the percentage of males hospitalized by diabetes to be insignificant. However, all predictors are deemed significant in the Poisson model. This shows how the GP model excels in controlling over-dispersion and producing precise estimates compared to the NB and Poisson models.

Based on the results in Table 3 from the GP regression models, we have provided prediction equations for the average diabetes count as follows:

$$log\left(\widehat\mu\right)=3.540+0.0616\ast\mathrm{pPLHIV}+0.2264\ast Pop-0.1507\ast GDP+0.0132\ast no.H.facilities+1.172\ast Urban-0.8480\ast Central-0.6483\ast Eastern-0.7265\ast Lake-0.8064\ast Southern-0.7467\ast Southern\;Highland$$

The antilogarithm of the prediction equation above gives the expected number of diabetes cases as given below:

$$\widehat\mu~=~\text{exp}(3.5400+0.0616\text{*pPLHIV}+0.2264\ast Pop-0.1507\ast GDP+0.0132\ast no.H.facilities+1.172\ast Urban-0.8480\ast Central-0.6483\ast Eastern-0.7265\ast Lake-0.8064\ast Southern-0.7467\ast Southern\;Highland)$$

**Table 4:** Information criteria

Model	Information Criteria				Pearson-$\boldsymbol{{\chi }^{2}}$	Pearson-$\boldsymbol{{\chi }^{2}/DF}$
	$\boldsymbol{-2logL}$	AIC	BIC	AICc		
GP	914.617	1857.234	1901.7	1859.826	332.3211	0.977
NB	917.775	1863.551	1908.017	1866.143	370.7664	1.20
Poisson	4583.633	9193.266	9234.556	9195.499	9574.291	58.380

Table 4 gives the goodness of fit results obtained using different information criteria. The results show that the GP model earns the smallest information criteria values, which means that it outperforms the NB and Poisson models in modeling the used data. Moreover, the results show a slight difference among values obtained by NB and GPD, which may indicate that these two models have slight differences when used to model over-dispersed data. However, the major difference between them is that the GP model is appropriate for modeling equal, over, and under-dispersed data, while the NB model is used for modeling over-dispersed data.

In Table 4, there are Pearson chi-square (Pearson-${\chi }^{2}$) and Pearson-${\chi }^{2}/DF$ values for the GP, NB, and Poisson models. A value of Pearson-${\chi }^{2}/DF$ greater than one means there is over-dispersion, and if it is exactly or close to one, it means over-dispersion is well controlled. The GP model has a value closest to one compared to the other models, making it the best choice for modeling over-dispersed diabetes count data.

## Discussion

This paper suggests utilizing the GP model to model socio-environmental and other risk factors associated with diabetes incidences in Tanzania mainland. The GP model’s performance was compared to that of NB and Poisson, as these three models are related. The NB model was obtained through a parametrization process called Poisson mixture gamma, which can model over-dispersed data that the standard Poisson model cannot. Additionally, the model can be reduced to the Poisson model when the dispersion parameter tends to infinity. Similarly, the GP model was obtained as a limit of the NB model and can model over, under, and equally dispersed count data. Similar to the NB model, the GP model can also be reduced to the Poisson model when its dispersion parameter equals zero. These models belong to the GLM category and are widely used in analyzing the relationship between a response variable that follows exponential families of distributions and one or more predictor variables. Linear models are a specific type of GLM with an identity link function [9, 41, 42]. The link function transforms the response variable to conform to the linear model assumption, connecting the mean of the response variable to a linear combination of predictor variables.

This study’s findings reveal that the unequal dominance of diabetes cases is associated with the type of council residence. Both descriptive and inferential analyses show that urban areas have more diabetes cases than rural areas probably due to the lifestyles in the two areas. Urban areas showed a strong positive contribution to diabetes cases, supporting that environmental factors, including urbanization, are a significant risk factor for diabetes and other NCDs [5, 43]. The findings also show a significant difference in the predicted log of diabetes cases among various zones. This indicates heterogeneity of the burden across socio-environmental attributes. The northern zone, the reference category, appears to have made a significant contribution, causing the projected log of diabetes counts in other zones to be adverse. The western zone was found to have a negligible association with the log of diabetes cases compared to the northern zone according to the GP model. This finding is related to those of Stanifer et al. [44], who observed that hypertension is environmentally clustered since people living together share social-cultural norms like eating habits, crops produced, and other behavioural patterns that affect NCDs.

The study also investigated the contribution of other factors in diabetes cases, and the findings revealed that an increased log of diabetes counts is also associated with the council’s population and the number of health facilities. On the contrary, GDP at market price is shown to be negatively associated with the log of diabetes counts. This indicates diabetes incidences are also more common in low-income societies. Several researchers have observed a high NCD rate in low- and middle-income countries (LMICs), which aligns with these findings [3]. On the other hand, the total number of patients who attended hospitals for HIV care is not associated with diabetes cases. This result differs from those obtained by Castilho et al. [45]. The percentage of male diabetes cases does not significantly relate to total diabetes cases in the councils. This factor is used to measure the contribution of sex to diabetes incidence, as other studies concluded that there is a higher prevalence of NCD cases among males than females in Africa [3]. Also, there is empirical evidence of a high economic burden among poor households in Tanzania caused by NCDs [46]. This study findings reveals dominance of diabetes incidences among councils with low GDP which may increase poverty contrary to Sustainable Development Goal 1.

The GP model performs better than both the NB and traditional Poisson regression models based on the log-likelihood value, AIC, BIC, AICc and Pearson-${\chi }^{2}$ values. The model achieves the lowest value among all information criteria, suggesting that GP is better at controlling over-dispersion among diabetes counts than its competitors. To determine the dispersion value of the data, one can also divide the Pearson chi-square value by its degree of freedom. This value should be close to or equal to 1 for equally dispersed data in the Poisson model. In this study, the value of Pearson-${\chi }^{2}/DF$ for the Poisson model is far greater than 1, indicating the presence of over-dispersion. The problem is well handled in the GP model.

## Conclusion

Considering the variability of count data when conducting statistical modeling is crucial. Ignoring this factor can lead to false estimates of the standard error, affecting the test statistic and p-value. It is crucial to examine the dispersion nature of the data to avoid incorrect inferences during statistical modeling of count data.

Hence, this study recommends the use of the GP model in modeling risk factors associated with disease count incidences, specifically in data collected among population subgroups with varying social and environmental characteristics. The model can accommodate count data collected in population subgroups with equal and unequal dispersion. The model is advantageous because it does not involve a difficult computation burden, it does not suffer from convergence issues and gives precise results compared to the most applied NB and Poisson models.

### Limitations of the study

The data in DHIS2 are recorded for very broad age groups which hinders further comparison regarding disease incidences. Additionally, the system does not include important patient information, which also limits model variables.

## Data Availability

The dataset that has been analyzed cannot be accessed by the public due to regulations regarding the usage and publication of health data in Tanzania. This study has obtained permission to use DHIS2 data but is strictly prohibited from sharing it. However, interested parties can request access to the data by submitting a proper request to the Tanzania Ministry of Health.

## Abbreviations

AIC: Akaike Information Criteria
AICc: Corrected Akaike Information Criteria
BIC: Bayesian Information Criteria
DF: Degrees of Freedom
DHIS2: District Health Information System 2
GDP: Gross Domestic Product
GLM: Generalized Linear Model
GP: Generalized Poisson
HIV: Human Immunodeficiency Virus
NB: Negative Binomial
NBS: National Bureau of Statistics
NCD: Non-Communicable Disease
SE: Standard Error
